# Teeth

**Published:** 1882-11

**Authors:** George A. Hendricks


### TEETH.

By GEORGE A. HENDRICKS, M. D.

[Curator of the Medical Museum of the University of Michigan.]

So many interesting facts are locked up within the tooth's hard casement, and so long and obstinately has it resisted the hammer and saw of the investigator, that it has long become an object of special study to the scientist. Give a tooth to a naturalist, and he, like the great Agassiz who when given a scale, drew a picture of the fish from which it was taken, can draw the picture of the animal that produced the tooth, even though the species be extinct a thousand years. The naturalist can tell if the animal fed upon grass; if he couchingly awaited the approach of a weaker animal upon which to feast; if it climbed the tree and gathered the nuts, or tunneled the ground to make its meal upon its juicy roots. Thus to the physiologist, on account of its intimate relation to the food and life of the animal, the tooth is of great interest; its many forms serve as a guide to the naturalist; from their convenience of position they can be easily examined in dead or living animals practically important as zoological features; their mineral composition renders them more durable than the other tissues of the body, which enable the palæontologist to determine the nature and habits of extinct species. Indeed, in no structure, is the law of *harmony of organs*, illustrated in a more striking manner than in the study of the teeth Even the internal structure of teeth is so peculiar in each group of animals, that it is possible to determine with precision the general structure of an animal merely by investigating the *smallest fragment* of a tooth under the microscope.

### WHAT ARE TEETH?

They are small hard bodies fixed into the bones or lining membrane of the mouth and throat, and perform the various offices in connection with the prehension and comminution of food. As such they are peculiar to *backboned* animals. They are not, as is generally supposed, developed from the bone, in which they are so firmly held, but they grow from the mucous membrane that lines the mouth. This membrane, was at one time in the youth of the animal, a part of the external skin with which it is now blended at the lips. If the growth of a hatching fish or the embryo of any animal be watched, it will be found that at one period of its existence, when its mouth is being developed, it has a distinct upper lip, but that the skin turns in over its under jaw. By the time the cavity of the mouth is truly formed, this membrane has become somewhat modified in appearance, but as its structure remains nearly the same as the external tegumen, a growth from either has about the same primary plan of development as a hair. A process grows down from one of the layers, (malpighian) of the membrane, into a depression which at the same time is produced for it in the tissue underneath. A small elevation (papilla) soon rises from the bottom of this depression, into the "down-growth." This papilla becomes the germ from which the growing structure will be developed, and the "down-growth" from the malpighian layer is changed to a simple sac or follicle enclosing the germ; in the one case the contents of this sac becomes a hair and in the other a tooth, according to situation.

In the case of the young fish the skin as it recedes into the mouth carries scale or spine follicles with it. From these follicles are developed modified spines. In the shark they become so very much larger than those on the outside that their identity is lost. Many fishes however have spines covering their bodies that in size and form closely resemble the teeth of the shark. Thus whether we study the development of the parts, or make comparison between adult forms, it is seen that the teeth of the shark correspond in mode of development to the teeth of other fishes, and these in turn to those of higher animals. Having shown that the teeth of fishes are highly developed spines, and that all teeth bear similar relation to the skin, it is easily seen why they are classed with the hair, nails, etc., "dermal appendages;" and why they are regarded as perfectly distinct from internal bony skeleton. Since all teeth are developed from the mucous membrane of the mouth, it must not be inferred that they have similar connections with the hard parts, or that they are at all attached to the jaws. In many fishes, and particularly in the sharks, they remain in the membrane, having no attachment to the bone; in others they fit more or less loosely into cavities of the jaw; while in some animals they become firmly united (anchylosed) to the bone. Teeth are not only attached to the jaws but they are found on the tongue, in the throat, and on plates of bone forming the roof of the mouth. This profuseness is confined to the fishes; on reptiles they are less widely distributed; while in mammals they are limited to the mafillans bones. In the lower orders teeth are very numerous and similar in form, in mammals they are fewer in number and adapted in form to various functions.

As the teeth develop, various forms of lime are deposited in the soft growth previously described. The hardening of these preexisting cells always proceeds from without inward and may continue until the whole tooth is completely solidified. In most cases it does not advance to this condition but leaves a soft mass, which is the "pulp" or "sensitive part" of the tooth, within a hard shell or dentine. When this pulp becomes almost completely enclosed by dentine, the tooth ceases to grow, as is the case with human teeth and all others that have roots. But this vascular mass may be continually reproduced at the base of the tooth, and as constantly converted into new dentine; in this case the tooth is of perpetual growth.

If a longitudinal section be taken of a human incisor, three distinct hard substances will be exposed. The enamel white and extremely hard covering for the exposed portion or *crown*; a relatively soft yellowish substance deposited on the *fang* or *root*, called "*cementum*" while the great mass of the tooth is seen to be made up of a bone-like material, the "*dentine*" which gives its characteristic form. The cavity within the dentine, which has been opened, contains the "pulp."

The above arrangement is true of all the human teeth and the "nippers" of most mammalia. But these hard parts do not always bear the same relation to one another. Examine the grinding surface of a horse's molar, and it will be found to be arranged in laminal more or less perpendicular to the surface. Those sharp ridges on enamel, the grooves are formed by the wearing away of the dentine and cementum. These plicated layers vary with the different families of ruminents. In one they run parallel with the long axis of the tooth, in another they are transverse.

In the order of gnawers or rodents the incisors have quite a different arrangement. The tooth is made up of a body of dentine with a plate of enamel laid on its front or anterior surface. The plate consists of several layers of varying density, the hardest being the most anterior. These teeth have always an oblique cutting surface, like a chisel, sloped backward from their short anterior margin.

### HOW MANY.

Some hints have been made, necessarily, regarding the number of teeth. In fishes where there is offered a most striking series of varieties, they range from 0 to 8. The sturgeon, whose mouth is nothing more than a protrusible sucker, is without teeth; the little sea-horse, common in aquarias, is also endentulus; the ray has its mouth filled with plates or prisms, having a tessellated arrangement; while the teeth which fill the mouth and throat of the pike are numberless. These are extremes between which there is every grade.

Among the reptiles the whole order chelonia are without teeth; toads are just as destitute; while frogs have their upper jaws supplied, their lower going without; salamanders and some lizards have their jaws filled, and throats also; in crocodiles and most serpents they are limited in number and confined to the jaw bones.

There are a few genera and species among mammals that are edentata. All true ant-eaters are strictly devoid of teeth; whales, prior to birth, have on the margin of both jaws a series of round rudimentary teeth, which are soon shed, being succeeded by whalebone plates,—the adult whale has no teeth; the elephant has never more than one whole tooth, or parts of two, for each side of the jaws, his front teeth are developed into enormous tusks; true ruminants have no incisors in their upper jaw, their entire number is thirty-two. The same number characterizes man and the apes, and is the average one for the class mammalia; but one hundred and ninety must be counted to number the teeth of the dolphin, the greatest number found in any mammal.

The reproduction and physiology will be considered in a future paper.

